# Modified-Dose Pembrolizumab and Prognostic Outcomes among Non-Small Cell Lung Cancer Patients: A Chart Review Study

**DOI:** 10.3390/ijerph19105999

**Published:** 2022-05-15

**Authors:** Sheng-Yin To, Li-Ting Kao, Jui-Hu Shih, I-Hsun Li, Tsai-Wang Huang, Chen-Liang Tsai, Chih-Feng Chian, Ching-Liang Ho, Ping-Ying Chang

**Affiliations:** 1 Department of Pharmacy Practice, Tri-Service General Hospital, Taipei 11490, Taiwan; ein060113@gmail.com (S.-Y.T.); kaoliting@gapps.ndmctsgh.edu.tw (L.-T.K.); jtlovehl@gmail.com (J.-H.S.); lhs01077@gmail.com (I.-H.L.); 2 School of Public Health, National Defense Medical Center, Taipei 11490, Taiwan; 3 School of Pharmacy, National Defense Medical Center, Taipei 11490, Taiwan; 4 Graduate Institute of Life Sciences, National Defense Medical Center, Taipei 11490, Taiwan; 5 Department of Pharmacology, National Defense Medical Center, Taipei 11490, Taiwan; 6 Division of Thoracic Surgery, Department of Surgery, Tri-Service General Hospital, National Defense Medical Center, Taipei 11490, Taiwan; chi-wang@yahoo.com.tw; 7 Division of Pulmonary and Critical Care Medicine, Department of Internal Medicine, Tri-Service General Hospital, National Defense Medical Center, Taipei 11490, Taiwan; doc10376@gmail.com (C.-L.T.); sonice3982@gmail.com (C.-F.C.); 8 Division of Hematology/Oncology, Department of Internal Medicine, Tri-Service General Hospital, National Defense Medical Center, Taipei 11490, Taiwan; hochingliang@yahoo.com.tw

**Keywords:** non-small cell lung cancer, pembrolizumab, neutrophil-to-lymphocyte ratio, low-dose, prognostic

## Abstract

The modified dose (MD) regimen of pembrolizumab (2 mg/kg or 100 mg every 3 weeks) is an alternative option to reduce the financial burden resulting from the extremely high cost of the standard dose (SD) regimen (200 mg every 3 weeks). However, the clinical effectiveness and prognostic outcomes have not been fully elucidated in real-word clinical practice. Sixty-four consecutive patients in Taiwan receiving pembrolizumab for advanced NSCLC between 2018 and 2020 were recruited in this study. Comparisons of overall survival (OS) and progression-free survival (PFS) were performed using Kaplan–Meier survival curves. Additionally, 12 predictors, including pembrolizumab regimen, dose, neutrophil-to-lymphocyte ratio (NLR), age, sex, histopathology, smoking history, ECOG PS, EGFR mutation, PD-L1 expression, distant metastases and treatment line, were analyzed in multivariable Cox models for predicting OS and PFS. The results showed that the MD group and the SD group had similar OS and PFS, especially in patients beyond first-line treatment or with a pretreatment NLR < 5. The NLR was the only independent factor associated with both OS (adjusted HR = 0.052; *p* = 0.010) and PFS (adjusted HR = 0.259; *p* = 0.021). The results of this study assure the clinical effectiveness of MD pembrolizumab and suggest that the pretreatment NLR could highlight patients who may benefit from MD pembrolizumab.

## 1. Introduction

Blocking antibodies that target programmed cell death 1 (PD-1) or programmed cell death protein ligand 1 (PD-L1) have been approved for the treatment of cancer and have significantly improved the outcomes of various advanced cancers, including non-small cell lung cancer (NSCLC) [1,2,3,4,5]. In 2015, pembrolizumab was the first anti-PD-1 antibody approved by the United States Food and Drug Administration (US FDA) for the treatment of advanced NSCLC [6]. Soon after, it became part of clinical practice and has been recommended for the first-line treatment of advanced NSCLC tumors with no driver gene mutation, such as epidermal growth factor receptor (EGFR) mutation or anaplastic lymphoma kinase (ALK) rearrangement, and those with a PD-L1 expression tumor proportion score (TPS) of ≥1% [7].

In contrast to conventional cytotoxic agents, the relationship between the dose and efficacy of pembrolizumab is not linear [8,9,10]. In the pivotal clinical trials, anti-tumor activity was observed at all doses (1–10 mg/kg) and schedules (every two or three weeks) [11]. In addition, multiple studies have demonstrated that there is no dose–response relationship between the effectiveness and safety of pembrolizumab [12,13,14,15]. Furthermore, another study found that there was similar exposure distribution in the pharmacokinetic simulations of a 2 mg/kg dose and a 200 mg fixed dose [16]. A fixed dose of 200 mg pembrolizumab (defined as the standard dose, SD) was the schedule approved by the US FDA for the treatment of patients with advanced NSCLC [17] and was used in the subsequent trials [18,19,20].

However, pembrolizumab was hampered by its extremely high cost [21]. Moreover, the SD was reported to lead to unnecessary dose and economic impacts in patients who were a standard weight [22,23,24]. Goldstein et al. showed that a pembrolizumab dose reduction strategy could save approximately 24.0% of the annual cost, compared with the cost of the 200 mg fixed dose [23]. Thus, physicians routinely use modified dose (MD) pembrolizumab (2 mg/kg or a fixed dose of 100 mg) for patients who cannot afford the financial burden in real-word clinical practice [24,25]. However, evidence on the real-word effectiveness and prognostic outcomes of the MD regimen remains limited. This study aimed to compare the clinical effectiveness of MD and SD pembrolizumab for the treatment of advanced NSCLC. In addition, we further assessed the utility of the pretreatment neutrophil-to-lymphocyte ratio (NLR) in determining MD and SD pembrolizumab benefit.

## 2. Materials and Methods

### 2.1. Study Population and Treatment

This was a retrospective chart review study that recruited all patients aged >20 years who received pembrolizumab for advanced NSCLC between 1 July 2018 and 31 December 2020 at a medical center in Taiwan. The index date was defined as the time of initiation of pembrolizumab for each patient. Patients who were in any clinical trial during the study period and those being treated in combination with other immune checkpoint inhibitors (ICIs) were excluded from this study. The eligible patients were further stratified into SD and MD groups. The SD group received pembrolizumab at 200 mg every three weeks, whereas the MD group received pembrolizumab at 2 mg/kg or 100 mg every three weeks. The definition of MD in this study was based on the comparison of clinical outcomes of pembrolizumab [5,24,26]. All patients below the standard dose were pooled in the MD analysis in this study. The study population comprised a total of 64 patients; 36 were in the MD group and 28 were in the SD group. 

The patients’ baseline demographics and clinical characteristics, including pembrolizumab regimen, dose, NLR, age, sex, histopathology, smoking history, Eastern Cooperative Oncology Group performance status (ECOG PS), EGFR mutation, PD-L1 expression, distant metastases and treatment line, were collected and considered as the confounders in the Cox regression models.

### 2.2. Outcome Measurements

A computed tomography scan was performed every three months as a routine clinical procedure. Clinical response to pembrolizumab was assessed by response evaluation criteria in solid tumors (version 1.1) [27,28]. Overall survival (OS) was the primary endpoint. Additionally, progression-free survival (PFS), overall response rate (ORR), disease control rate (DCR) and immune-related adverse events (irAEs) were identified as the secondary endpoints. OS was defined as the duration from the initiation of pembrolizumab to death from any cause or the last follow-up. PFS was measured from the time of treatment initiation to disease progression, death from any cause or the last follow-up, whichever occurred first. For OS, this study censored the data for surviving or missing patients at the time they were last known to be alive. For PFS, our study censored the data for patients surviving without disease progression or missing at the time of the last tumor assessment. ORR was defined as the percentage of patients with a confirmed partial response (PR) or complete response (CR). DCR was defined as the sum of PR, CR and stable disease. All subjects were followed from the index date until the occurrence of events or until March 31, 2021 (when the study finished). The presence of irAEs was based on an assessment by the treating physician, and the irAEs were graded according to the Common Terminology Criteria for Adverse Events, version 5.0.

According to previously reported data showing that pretreatment NLR was an independent prognostic indicator in patients with advanced NSCLC treated with anti-PD1 antibodies [29,30], baseline pretreatment peripheral blood cell counts were collected. The NLR was further calculated as the neutrophil count divided by the lymphocyte count.

### 2.3. Statistical Analysis

The clinical and disease characteristics were determined as median and range for continuous variables and as frequency and percentage for categorical variables. The Wilcoxon rank sum test was used to compare the continuous variables between groups. A chi-square test or Fisher’s exact test was used for categorical variables. The comparisons of PFS and OS between groups were further analyzed by Kaplan–Meier survival curves with log rank tests. A Cox proportional hazards regression model was used to consider the related factors affecting OS and PFS. Hazard ratios (HRs) and 95% confidence intervals (CIs) for the OS and PS were calculated. For testing the proportional hazard assumption, we checked whether the independent variables met the proportional assumption using a log-minus-log (LML) plot; the LML plot resulted in parallel curves. The models used in this study met the proportional hazards assumption. A logistic regression model was used to consider the related factors affecting ORR. A two-tailed p value of <0.05 was considered statistically significant. Analyses were carried out using IBM SPSS Version 22.

### 2.4. Sample Size

The sample size and power calculations were performed with G*Power [31]. In our study, a sample size of 64 total evaluable patients was calculated to provide 90% power to detect a difference of 5% in the tumor response between the two groups (two-sided α = 0.05).

## 3. Results

### 3.1. Patient Characteristics

A total of 64 patients with advanced NSCLC were included in this study. Thirty-six patients (56.3%) were in the MD group and 28 patients (43.8%) were in the SD group. The baseline demographics and clinical characteristics of each group are presented in Table 1. The median age was 62 years; 57.8% of participants were men and 46.9% were current or ex-smokers. In the whole cohort, 84.4% had adenocarcinoma, and most of the patients did not have driver mutations. Of the 55 (85.9%) patients who were tested for PD-L1, 31 (48.4%) had a PD-L1 TPS ≥50%. The median pretreatment NLR was 5.3 (1.3–35.5, data not shown). The two groups were similar in terms of age, sex, smoking history, ECOG PS, histologic subtype, EGFR and ALK tumor mutation status, PD-L1 TPS score and pretreatment NLR. Body weight was significantly higher in the SD group than in the MD group (*p* = 0.012).

The median pembrolizumab dose was significantly lower in the MD group (MD vs. SD: 1.8 mg/kg vs. 3.3 mg/kg; *p* < 0.001). No patients in the SD group received <2 mg/kg pembrolizumab. On the other hand, 63.9% patients in the MD group received <2 mg/kg pembrolizumab. Pembrolizumab was prescribed as first-line therapy in 25 (39.1%) patients and beyond first-line therapy in 39 (60.9%) patients. Compared with the MD group, the SD group had a significantly higher percentage of patients who received monotherapy (41.7% vs. 71.4%, *p* = 0.005) and first-line treatment (27.8% vs. 53.6%, *p* = 0.015). The median duration of SD treatment was 5.0 months and the median duration of MD treatment was 2.3months (*p* = 0.018).

### 3.2. OS and PFS between MD and SD Groups

**Figure 1:**
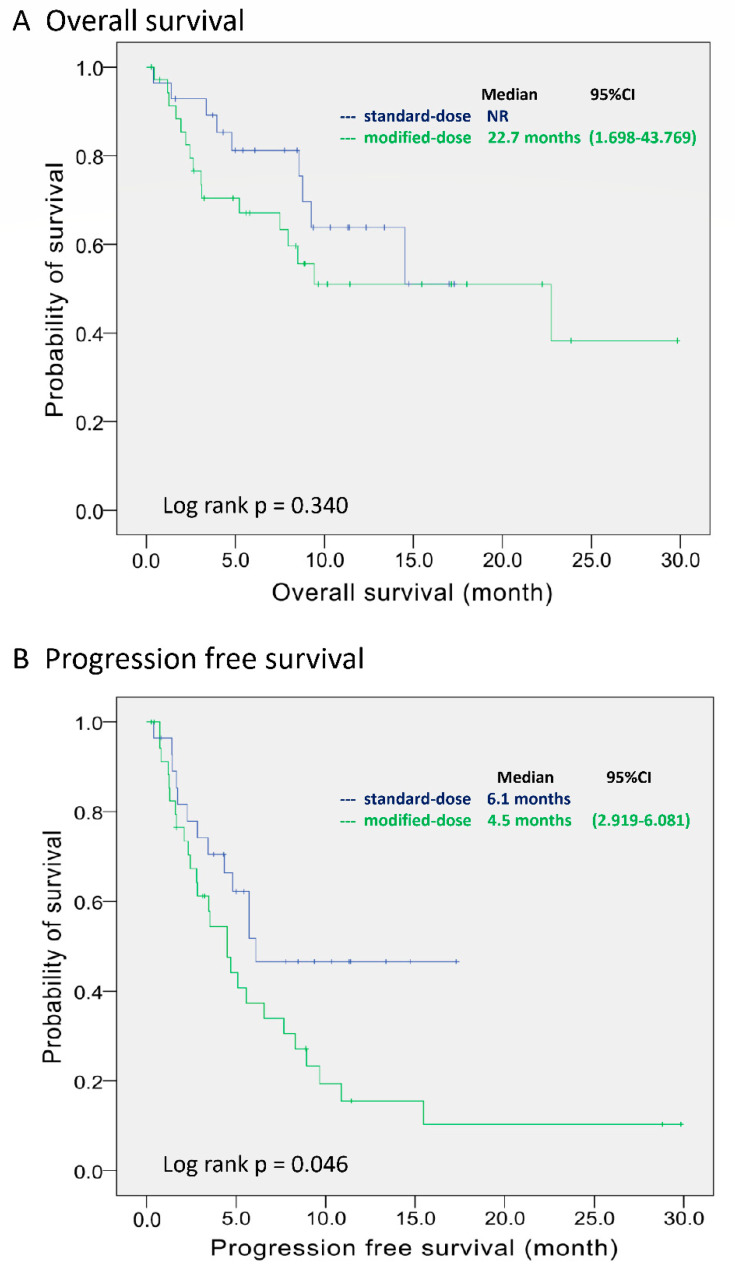
(**A**) Overall survival of patients treated with standard-dose pembrolizumab and modified-dose pembrolizumab. (**B**) Progression-free survival of patients treated with standard-dose pembrolizumab and modified-dose pembrolizumab. Abbreviations: OS—overall survival; PFS—progression-free survival; NR—not reached.

**Figure 2:**
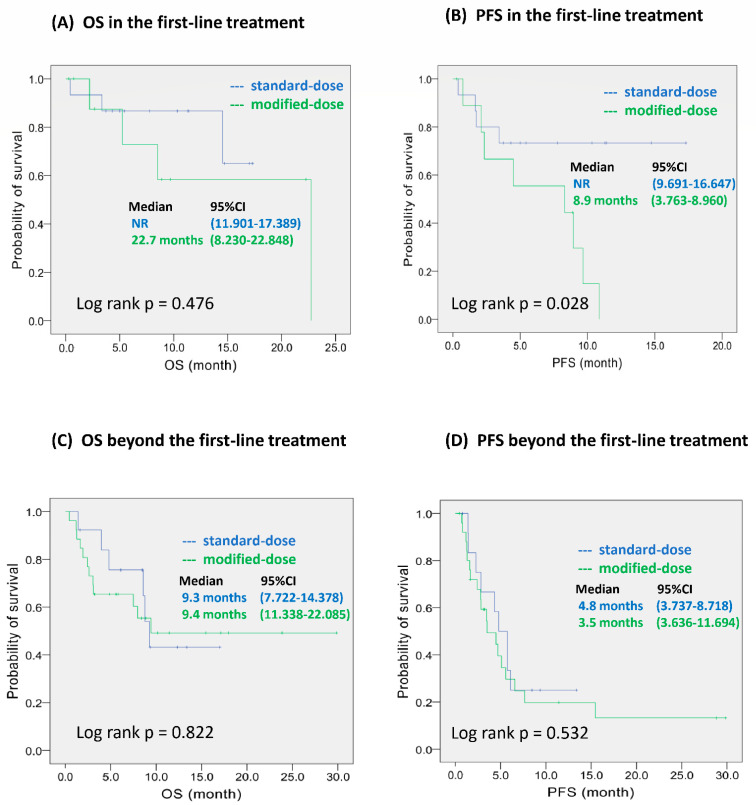
Kaplan–Meier survival analyses by risk group and dose of pembrolizumab. (**A**,**B**): OS and PFS in first-line treatment. (**C**,**D**): OS and PFS beyond first-line treatment. Abbreviations: OS—overall survival; PFS—progression-free survival; NR—not reached.

The median follow-up time was 7.9 months (range: 0.3–29.8 months) for all patients, with no difference between the MD and SD groups (6.7 months vs. 8.5 months, respectively, *p* = 0.457). As shown in Figure 1, there was no difference in OS between groups; however, PFS was significantly shorter in the MD group than in the SD group (median PFS: 4.5 months vs. 6.1 months; *p* = 0.046). When pembrolizumab was used as first-line treatment, there was no difference in OS between the groups (Figure 2A); however, PFS was significantly shorter in the MD group than in the SD group (*p* = 0.028; Figure 2B). When pembrolizumab was used beyond first-line treatment, there was no difference in OS (9.4 months vs. 9.3 months; *p* = 0.822) and PFS (3.5 months vs. 4.8 months; *p* = 0.532) between the two groups (Figure 2C,D).

**Figure 3:**
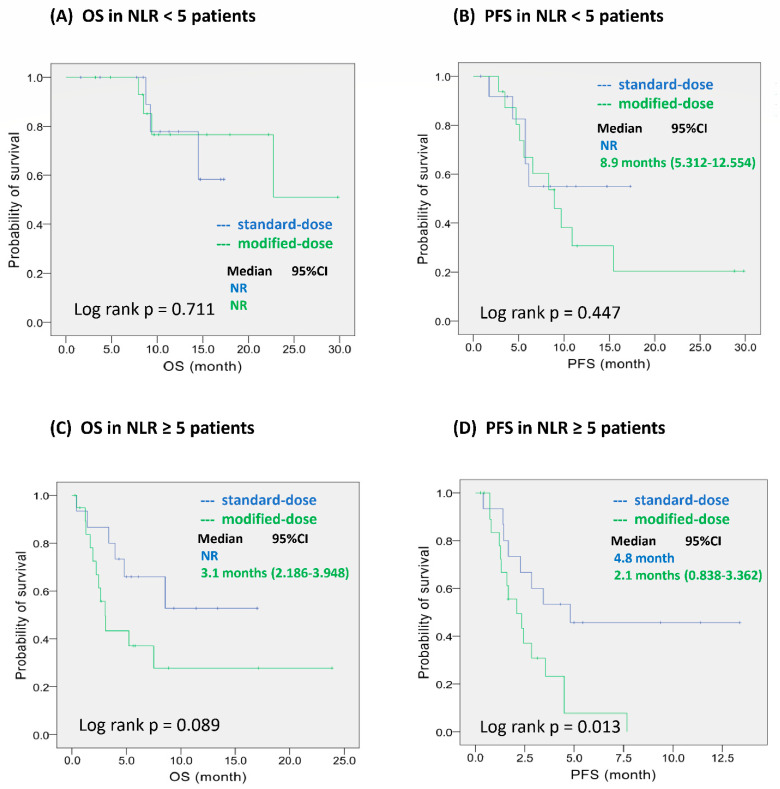
Kaplan–Meier survival analyses by risk group and dose of pembrolizumab. (**A**,**B**): OS and PFS in low NLR group (NLR < 5). (**C**,**D**): OS and PFS in high NLR group (NLR ≥ 5). Abbreviations: OS—overall survival; PFS—progression-free survival; NR—not reached.

Based on the median pretreatment NLR cut-off value of 5, we performed a subgroup analysis of the low NLR (NLR < 5) and high NLR groups (NLR ≥ 5). In patients with a low NLR (Figure 3A,B), there was no difference between the MD and SD groups in terms of OS (*p* = 0.711) and PFS (*p* = 0.447). In patients with a high NLR, there was no difference in OS between the two groups (*p* = 0.089, Figure 3C); however, PFS was significantly shorter in the MD group than in the SD group (2.1 months vs. 4.8 months; *p* = 0.013; Figure 3D).

### 3.3. Results of the Regression Model

Table 2 shows the univariable and multivariable Cox proportional hazards analyses. In the univariable Cox proportional hazards analysis, the factors associated with OS were pretreatment NLR (*p* = 0.001) and ECOG PS (*p* = 0.007), while the factors associated with PFS included pembrolizumab dose per body weight (*p* = 0.048), pretreatment NLR (*p* = 0.001) and ECOG PS (*p* = 0.044). The multivariable Cox proportional hazards models indicated that the independent factors related to OS were pretreatment NLR (adjusted HR = 0.052; 95% CI: 0.006–0.489; *p* = 0.010) and PD-L1 expression (adjusted HR = 0.218; 95% CI: 0.061–0.782; *p* = 0.019), while those for PFS were pretreatment NLR (adjusted HR = 0.259; 95% CI: 0.082–0.816; *p* = 0.021) and adenocarcinoma histology (adjusted HR = 0.052; 95% CI: 0.003–0.832; *p* = 0.037). In terms of ORR, after considering many potential confounders, pembrolizumab dose per body weight was found to be independently associated with ORR (adjusted OR = 1.867; 95% CI: 1.067–3.265; *p* = 0.029).

### 3.4. Tumor Response between MD and SD Groups

Table 3 shows the tumor response in the MD and SD groups. The ORR was significantly lower in the MD group than in the SD group (13.9% vs. 53.6%; *p* = 0.001); however, the DCR was similar between the two groups (63.9% vs. 78.6%; *p* = 0.202). When pembrolizumab was used as first-line treatment, the ORR tended to be lower in the MD group than in the SD group (30% vs. 66.7%; *p* = 0.086), and there was no difference in the DCR between the two groups (MD group 70% vs. SD group 80%; *p* = 0.517). When pembrolizumab was used beyond first-line treatment, there was no difference in the DCR between the two groups; however, the ORR was significantly lower in the MD group than in the SD group (7.7% vs. 38.5%; *p* = 0.033).

In patients with a low NLR, the ORR tended to be lower in the MD group than in the SD group (25% vs. 61.5%; *p* = 0.067), and there was no difference in the DCR between the two groups (87.5% vs. 84.6%; *p* = 1.0). In patients with a high NLR, there was no difference in the DCR between the two groups (45% vs. 73.3%; *p* = 0.167); however, the ORR was significantly lower in the MD group than in the SD group (5.0% vs. 46.7%; *p* = 0.011).

### 3.5. Toxicities

In the overall study population, thirteen patients (20.3%) developed any grade of immune related adverse effects (irAEs) and three patients (5%) discontinued treatment due to toxicities. Compared to the MD group, any grade of irAEs were significantly higher in SD group (11% vs. 32%; *p* = 0.038) and there was no difference in the rates of Grade 3/4 irAEs between the two groups (2.8% vs. 7.1%; *p* = 0.577).

## 4. Discussion

The results of our study showed that the MD and SD treatments had similar clinical effectiveness, especially in patients treated beyond first-line therapy or with an NLR < 5. In addition, the incidence of any grade of irAEs was significantly lower in the MD group than the SD group. We also demonstrated that the pretreatment NLR was the most important independent factor associated with survival results, including OS and PFS. To the best of our knowledge, this is the first study to assess the utility of the pretreatment NLR in determining MD pembrolizumab benefit in a real-world setting. Our study further highlights that pretreatment NLR could be used as a prognostic tool among patients who have decided to use pembrolizumab for advanced NSCLC

It is noteworthy that SD pembrolizumab as a first-line treatment was more effective in PFS than first-line MD pembrolizumab. Furthermore, MD pembrolizumab for first-line treatment showed a more than two-fold increase in both median PFS and OS when compared to MD pembrolizumab beyond first-line treatment (OS: 22.7 months versus 9.4 months and PFS: 8.9 months versus 3.5 months, respectively). In keynote-010, the Cox proportional hazards model for OS showed that high dose pembrolizumab could decrease the risk of death by 17% (HR for 2 mg/kg vs. 10 mg/kg: 1.17; 95% CI: 0.94–1.45), though the relevant findings did not reach statistical significance. In addition, a previous meta-analysis reported that first-line immunotherapy had better effectiveness than immunotherapy for use as a second-line treatment [32]. Accordingly, MD pembrolizumab seems to be a good option to use in beyond first-line treatment. In addition, SD pembrolizumab could be a protective factor for tumor response in first-line treatment. We believe that this study provides some evidence for clinicians and patients who wish to select MD pembrolizumab as an alternative choice.

To date, increasing studies have indicated that MD anti-PD-1 treatments may be important options for real-world practice settings to reduce the financial burden resulting from the extremely high cost of the SD regimen without compromising clinical outcomes. In 2018, Yoo SH et al. demonstrated that MD nivolumab (20 or 100 mg every three weeks) was as effective as SD nivolumab (3 mg/kg every two weeks) in advanced NSCLC [33], with no significant differences in OS, PFS, ORR and DCR. In 2021, Low JL et al. found that there was no significant difference in PFS, OS, ORR and DCR between MD (100 mg every three week) and SD pembrolizumab when used as monotherapy or in combination with chemotherapy [24]. The findings of these studies are inconsistent with our research, which observed a short PFS and low ORR in the MD group. In a prior study [24], 80% of the patients received pembrolizumab as first-line treatment. In addition, the MD group had significantly more patients with a PD-L1 TPS score ≥50% (Pem100: 68% vs. Pem200: 39%; *p* = 0.005). As mentioned above, first-line immunotherapy seems to be more effective than beyond first-line treatment, and it is well known that patients with a PD-L1 TPS score ≥50% can potentially account for a great deal of the favorable clinical outcomes. In our present study, the proportion of patients with a PD-L1 TPS score ≥50% was similar between the MD and SD groups (38.9% vs. 60.7%; *p* = 0.117). The aforementioned results may explain the shorter PFS and lower ORR in our MD group.

The pretreatment NLR is known as a prognostic predictor in patients with NSCLC receiving systemic therapy, including ICIs [34,35,36,37]. In our study, the pretreatment NLR was the only independent prognostic factor associated with both OS and PFS. Notably, PFS was relatively short and ORR was relatively low after the administration of MD pembrolizumab in patients with a high pretreatment NLR. In patients with cancer, the inflammation process has been suggested as the reason for immune resistance, which may promote tumor growth and invasion [38,39,40]. The inflammatory cells of the tumor microenvironment may break down the adaptive immune responses and block the response to anti-tumor treatment [40,41]. Our study confirmed that a high pretreatment NLR was an important factor for poor prognosis, and SD pembrolizumab may be used to improve the treatment outcome.

Our study had several limitations. First, due to the observational study design, some confounders might affect our findings. Nevertheless, we carried out relevant analyses and assured that there was no significant difference in the baseline characteristics between the two groups. Secondly, some researchers might criticize the limited sample size in this study. The small sample size is a common limitation in modified-dose immunotherapy studies [24,33] because of the economic burden on healthcare systems and patients [25]. However, we already evaluated the statistical power in our study to estimate the impact of sample size. The statistic power (>0.9) was sufficient to detect the relevant findings and avoid errors in this study. Despite these limitations, our study demonstrated the clinical effectiveness of MD pembrolizumab. Maintenance pembrolizumab is suggested when the patient is responsive or has stable disease after initial pembrolizumab monotherapy. However, financial burden should be considered if the cost is not reimbursed by health insurance. In such situations, the MD regimen with personalized weight-based dosing or a fixed dose of 100 mg tri-weekly could be an alternative choice.

## 5. Conclusions

This study demonstrated that MD and SD pembrolizumab had similar clinical effectiveness, especially in those patients receiving beyond first-line treatment or with a low NLR. However, the use of SD pembrolizumab could increase ORR and improve survival results in patients with a high NLR. Pretreatment risk classification using NLR may help identify patients who might benefit from MD pembrolizumab. Further studies are needed to clarify the potential predictive and prognostic indicators in patients receiving MD pembrolizumab.

## Figures and Tables

**Table 1:** Baseline demographic and clinical characteristics.

	Total (n = 64)	Modified Dose (n = 36)	Standard Dose (n = 28)	*p* Value
Age (years)				0.129
<65	39 (60.9)	19 (52.8)	20 (71.4)	
≥65	25 (39.1)	17 (47.2)	8 (28.6)	
Sex				0.355
Male	37 (57.8)	19 (52.8)	18 (64.3)	
Female	27 (42.2)	17 (47.2)	10 (35.7)	
Brain metastases				0.435
Yes	24 (37.5)	12 (33.3)	12 (42.9)	
No	40 (62.5)	24 (66.7)	16 (57.1)	
Histologic subtype				0.163
Adenocarcinoma	54 (84.4)	28 (77.8)	26 (92.9)	
Squamous cell carcinoma	8 (12.5)	7 (19.4)	1 (3.6)	
Other *	2 (3.1)	1 (2.8)	1 (3.6)	
Smoking history				0.95
Current or ex-smoker	30 (46.9)	17 (47.2)	14 (46.4)	
Never smoked	34 (53.1)	19 (52.8)	15 (53.6)	
ECOG PS				0.748
0–1	47 (73.4)	27 (75)	20 (71.4)	
≥2	17 (26.6)	9 (25)	8 (28.6)	
EGFR mutation				0.076
Yes	17 (26.6)	10 (27.8)	7 (25)	
No	38 (59.4)	18 (50)	20 (71.4)	
Unknown	9 (14.1)	8 (22.2)	1 (3.6)	
ALK rearrangement				0.935
Yes	2 (3.1)	1 (2.8)	1 (3.6)	
No	47 (73.4)	26 (72.2)	21 (75)	
Unknown	15 (23.4)	9 (25)	6 (21.4)	
PD-L1 (TPS score)				0.117
<1%	15 (23.4)	12 (33.3)	3 (10.7)	
1–49%	9 (14.1)	6 (16.7)	3 (10.7)	
≥50%	31 (48.4)	14 (38.9)	17 (60.7)	
Unknown	9 (14.1)	4 (11.1)	5 (17.9)	
Pretreatment NLR				0.874
<5	29 (45.3)	16 (44.4)	13 (46.4)	
≥5	35 (54.7)	20 (55.6)	15 (53.6)	
Dose/kg of pembrolizumab	2.4 (0.8–4.4)	1.8 (0.8–4.4)	3.3 (2.3–4.0)	<0.001
Line of treatment				0.015
First	25 (39.1)	10 (27.8)	15 (53.6)	
Second	15 (23.4)	7 (19.4)	8 (28.6)	
Third or beyond	24 (37.5)	19 (52.8)	5 (17.9)	
Partner drug				0.005
Monotherapy	35 (54.7)	15 (41.7)	20 (71.4)	
Combined CT ^a^	26 (40.6)	21 (58.3)	5 (17.9)	
Combined TKI ^b^	3 (4.7)	0	3 (10.7)	
Treatment duration (months)	2.9 (<0.1–17.3)	2.3 (<0.1–15.5)	5.0 (<0.1–17.3)	0.018

^a^ Combined chemotherapy (CT): platinum doublet (n = 20), pemetrexed (n = 2), docetaxel (n = 2) or navelbine (n = 2). ^b^ Combined tyrosine kinase inhibitors (TKIs): first generation TKI (n = 2) or third generation TKI (n = 1). * Other histological subtypes consist of one pleomorphic-like carcinoma and one poorly differentiated carcinoma. Abbreviations: ECOG—Eastern Cooperative Oncology Group; PS—performance status; EGFR—epidermal growth factor receptor; ALK—anaplastic lymphoma kinase; PD-L1—programmed death-ligand 1; TPS—tumor proportion score; NLR—neutrophil lymphocyte ratio.

**Table 2:** Univariable and multivariable Cox regression analysis for cancer-specific survival and various clinicopathologic factors.

	OS				PFS			
	Univariable		Multivariable		Univariable		Multivariable	
	HR (95% CI)	*p* Value	HR (95% CI)	*p* Value	HR (95% CI)	*p* Value	HR (95% CI)	*p* Value
Standard dose (ref: modified)	0.670 (0.293–1.533)	0.343	0.591 (0.059–5.954)	0.656	0.517 (0.266–1.004)	0.051	0.642 (0.105–3.927)	0.631
Dose (mg/kg)	0.790 (0.485–1.289)	0.346	0.677 (0.158–2.898)	0.599	0.687 (0.474–0.997)	0.048	0.543 (0.191–1.549)	0.254
Pretreatment NLR <5 (ref: NLR ≥ 5)	0.231 (0.095–0.566)	0.001	0.052 (0.006–0.489)	0.010	0.309 (0.158–0.604)	0.001	0.259 (0.082–0.816)	0.021
PD-L1 positive (ref: negative)	0.467 (0.190–1.151)	0.098	0.218 (0.061–0.782)	0.019	0.560 (0.271–1.158)	0.118	0.425 (0.160–1.130)	0.086
ECOG PS 0-1 (ref: ≥2)	0.33 (0.147–0.740)	0.007	0.444 (0.108–1.818)	0.259	0.495 (0.250–0.980)	0.044	0.411 (0.126–1.340)	0.141
ADC (ref: SqCC)	1.760 (0.412–7.524)	0.446	0.062 (0.002–1.652)	0.097	0.911 (0.380–2.188)	0.836	0.052 (0.003–0.832)	0.037
EGFR mutation (ref: no)	1.224 (0.494–3.033)	0.663	1.665 (0.356–7.788)	0.517	1.975 (0.967–4.036)	0.062	1.154 (0.381–3.494)	0.800
Age < 65 (ref: ≥65)	0.674 (0.307–1.482)	0.327	0.296 (0.070–1.248)	0.097	0.972 (0.515–1.835)	0.909	1.463 (0.503–4.253)	0.485
Male (ref: Female)	0.877 (0.397–1.936)	0.745	0.696 (0.129–3.749)	0.673	0.969 (0.517–1.816)	0.922	0.980 (0.285–3.364)	0.974
Smoking (ref: non)	1.291 (0.382–2.860)	0.530	3.161 (0.382–26.188)	0.286	0.645 (0.344–1.210)	0.172	0.450 (0.112–1.806)	0.260
Brain metastases (ref: no)	0.969 (0.427–2.197)	0.939	0.713 (0.173–2.938)	0.639	1.269 (0.665–2.422)	0.469	1.191 (0.461–3.072)	0.718
First-line therapy (ref: ≥2)	0.596 (0.248–1.430)	0.247	1.119 (0.166–7.552)	0.908	0.520 (0.263–1.028)	0.060	1.062 (0.312–3.616)	0.923

Abbreviations: OS—overall survival; PFS—progression-free survival; HR—hazard ratio; CI—confidence interval; ref—reference; NLR—neutrophil lymphocyte ratio; PD-L1—programmed death-ligand 1; ECOG—Eastern Cooperative Oncology Group; PS—performance status; ADC—adenocarcinoma; SqCC—squamous cell carcinoma; EGFR—epidermal growth factor receptor.

**Table 3:** Tumor response outcomes of modified-dose and standard-dose groups.

	Best Overall Response, No. (%)						ORR *		DCR ^†^
	CR	PR	SD	PD	NE	No. (%)	*p* Value	No. (%)	*p* Value
All patients (n = 64)									
Modified-dose group (n = 36)	0	5 (13.9)	18 (50)	9 (25)	4 (11.1)	5 (13.9)	0.001	23 (63.9)	0.202
Standard-dose group (n = 28)	1 (3.6)	14 (50)	7 (25)	4 (14.3)	2 (7.1)	15 (53.6)		22 (78.6)	
First-line treatment (n = 25)									
Modified-dose group (n = 10)	0	3 (30)	4 (40)	2 (20)	1 (10)	3 (30)	0.086	7 (70)	0.517
Standard-dose group (n = 15)	1 (6.7)	9 (60)	2 (13.3)	2 (13.3)	1 (6.7)	10 (66.7)		12 (80)	
Beyond first-line treatment (n = 39)									
Modified-dose group (n = 26)	0	2 (7.7)	14 (53.8)	7 (26.9)	3 (11.5)	2 (7.7)	0.033	16 (61.5)	0.324
Standard-dose group (n = 13)	0	5 (38.5)	5 (38.5)	2 (15.4)	1 (7.7)	5 (38.5)		10 (77)	
Low NLR (n = 29)									
Modified-dose group (n = 16)	0	4 (25)	10 (62.5)	1 (6.3)	1 (6.3)	4 (25)	0.067	14 (87.5)	1
Standard-dose group (n = 13)	1 (7.7)	7 (53.8)	3 (23.1)	2 (15.4)	0	8 (61.5)		11 (84.6)	
High NLR (n = 35)									
Modified-dose group (n = 20)	0	1 (5)	8 (40)	8 (40)	3 (15)	1 (5)	0.011	9 (45)	0.167
Standard-dose group (n = 15)	0	7 (46.7)	4 (26.7)	2 (13.3)	2 (13.3)	7 (46.7)		11 (73.4)	

* Objective response rate was calculated as the summation of CRs and PRs. ^†^ Disease control rate was calculated as the summation of CRs, PRs and SDs. Abbreviations: CR—complete response; PR—partial response; SD—stable disease; PD—progressive disease; NE—not evaluable; ORR—objective response rate; DCR—disease control rate; NLR—neutrophil lymphocyte ratio.

## Data Availability

The data presented in this study are available upon request from the corresponding author.

## References

[1] Herbst R.S., Baas P., Kim D.W., Felip E., Perez-Gracia J.L., Han J.Y., Molina J., Kim J.H., Arvis C.D., Ahn M.J. (2016). Pembrolizumab versus docetaxel for previously treated, PD-L1-positive, advanced non-small-cell lung cancer (KEYNOTE-010): A randomised controlled trial. Lancet.

[2] Horn L., Spigel D.R., Vokes E.E., Holgado E., Ready N., Steins M., Poddubskaya E., Borghaei H., Felip E., Paz-Ares L. (2017). Nivolumab Versus Docetaxel in Previously Treated Patients With Advanced Non-Small-Cell Lung Cancer: Two-Year Outcomes From Two Randomized, Open-Label, Phase III Trials (CheckMate 017 and CheckMate 057). J. Clin. Oncol..

[3] Eggermont A.M., Blank C.U., Mandala M., Long G.V., Atkinson V.G., Dalle S., Haydon A.M., Meshcheryakov A., Khattak A., Carlino M.S. (2020). Longer follow-up confirms recurrence-free survival benefit of adjuvant pembrolizumab in high-risk stage III melanoma: Updated results from the EORTC 1325-MG/KEYNOTE-054 trial. J. Clin. Oncol..

[4] Cortes J., Cescon D.W., Rugo H.S., Nowecki Z., Im S.-A., Yusof M.M., Gallardo C., Lipatov O., Barrios C.H., Holgado E. (2020). Pembrolizumab plus chemotherapy versus placebo plus chemotherapy for previously untreated locally recurrent inoperable or metastatic triple-negative breast cancer (KEYNOTE-355): A randomised, placebo-controlled, double-blind, phase 3 clinical trial. Lancet.

[5] Mehra R., Seiwert T.Y., Gupta S., Weiss J., Gluck I., Eder J.P., Burtness B., Tahara M., Keam B., Kang H. (2018). Efficacy and safety of pembrolizumab in recurrent/metastatic head and neck squamous cell carcinoma: Pooled analyses after long-term follow-up in KEYNOTE-012. Br. J. Cancer.

[6] Pai-Scherf L., Blumenthal G.M., Li H., Subramaniam S., Mishra-Kalyani P.S., He K., Zhao H., Yu J., Paciga M., Goldberg K.B. (2017). FDA Approval Summary: Pembrolizumab for Treatment of Metastatic Non-Small Cell Lung Cancer: First-Line Therapy and Beyond. Oncologist.

[7] Ettinger D.S., Wood D.E., Aisner D.L., Akerley W., Bauman J.R., Bharat A., Bruno D.S., Chang J.Y., Chirieac L.R., D’Amico T.A. (2021). NCCN Guidelines Insights: Non-Small Cell Lung Cancer, Version 2.2021. J. Natl. Compr. Cancer Netw..

[8] Elassaiss-Schaap J., Rossenu S., Lindauer A., Kang S.P., de Greef R., Sachs J.R., de Alwis D.P. (2017). Using Model-Based “Learn and Confirm” to Reveal the Pharmacokinetics-Pharmacodynamics Relationship of Pembrolizumab in the KEYNOTE-001 Trial. CPT Pharmacomet. Syst. Pharmacol..

[9] Chatterjee M., Turner D.C., Felip E., Lena H., Cappuzzo F., Horn L., Garon E.B., Hui R., Arkenau H.T., Gubens M.A. (2016). Systematic evaluation of pembrolizumab dosing in patients with advanced non-small-cell lung cancer. Ann. Oncol..

[10] Patnaik A., Kang S.P., Rasco D., Papadopoulos K.P., Elassaiss-Schaap J., Beeram M., Drengler R., Chen C., Smith L., Espino G. (2015). Phase I Study of Pembrolizumab (MK-3475; Anti-PD-1 Monoclonal Antibody) in Patients with Advanced Solid Tumors. Clin. Cancer Res..

[11] Garon E.B., Rizvi N.A., Hui R., Leighl N., Balmanoukian A.S., Eder J.P., Patnaik A., Aggarwal C., Gubens M., Horn L. (2015). Pembrolizumab for the treatment of non-small-cell lung cancer. N. Engl. J. Med..

[12] Hui R., Garon E.B., Goldman J.W., Leighl N.B., Hellmann M.D., Patnaik A., Gandhi L., Eder J.P., Ahn M.J., Horn L. (2017). Pembrolizumab as first-line therapy for patients with PD-L1-positive advanced non-small cell lung cancer: A phase 1 trial. Ann. Oncol..

[13] Kang S., Gergich K., Lubiniecki G., de Alwis D., Chen C., Tice M., Rubin E. (2017). Pembrolizumab KEYNOTE-001: An adaptive study leading to accelerated approval for two indications and a companion diagnostic. Ann. Oncol..

[14] Leighl N.B., Hellmann M.D., Hui R., Carcereny E., Felip E., Ahn M.-J., Eder J.P., Balmanoukian A.S., Aggarwal C., Horn L. (2019). Pembrolizumab in patients with advanced non-small-cell lung cancer (KEYNOTE-001): 3-year results from an open-label, phase 1 study. Lancet Respir. Med..

[15] Lim S.H., Sun J.-M., Lee S.-H., Ahn J.S., Park K., Ahn M.-J. (2016). Pembrolizumab for the treatment of non-small cell lung cancer. Expert Opin. Biol. Ther..

[16] Freshwater T., Kondic A., Ahamadi M., Li C.H., de Greef R., de Alwis D., Stone J.A. (2017). Evaluation of dosing strategy for pembrolizumab for oncology indications. J. Immunother. Cancer.

[17] (2021). Keytruda (Pembrolizumab) Package Insert.

[18] Reck M., Rodriguez-Abreu D., Robinson A.G., Hui R., Csoszi T., Fulop A., Gottfried M., Peled N., Tafreshi A., Cuffe S. (2016). Pembrolizumab versus Chemotherapy for PD-L1-Positive Non-Small-Cell Lung Cancer. N. Engl. J. Med..

[19] Mok T.S.K., Wu Y.L., Kudaba I., Kowalski D.M., Cho B.C., Turna H.Z., Castro G., Srimuninnimit V., Laktionov K.K., Bondarenko I. (2019). Pembrolizumab versus chemotherapy for previously untreated, PD-L1-expressing, locally advanced or metastatic non-small-cell lung cancer (KEYNOTE-042): A randomised, open-label, controlled, phase 3 trial. Lancet.

[20] Gandhi L., Rodriguez-Abreu D., Gadgeel S., Esteban E., Felip E., De Angelis F., Domine M., Clingan P., Hochmair M.J., Powell S.F. (2018). Pembrolizumab plus Chemotherapy in Metastatic Non-Small-Cell Lung Cancer. N. Engl. J. Med..

[21] Loong H.H., Wong C.K., Leung L.K.S., Dhankhar P., Insinga R.P., Chandwani S., Hsu D.C., Lee M.Y., Huang M., Pellissier J. (2020). Cost effectiveness of PD-L1-based test-and-treat strategy with pembrolizumab as the first-line treatment for metastatic NSCLC in Hong Kong. Pharm.-Open.

[22] Pires V., Ribeiro M., Gouveia A. (2019). 2SPD-013 Economic impact of the use of flat dose vs. personalised dose of pembrolizumab..

[23] Goldstein D.A., Gordon N., Davidescu M., Leshno M., Steuer C.E., Patel N., Stemmer S.M., Zer A. (2017). A Phamacoeconomic Analysis of Personalized Dosing vs. Fixed Dosing of Pembrolizumab in Firstline PD-L1-Positive Non-Small Cell Lung Cancer. J. Natl. Cancer Inst..

[24] Low J.L., Huang Y., Sooi K., Ang Y., Chan Z.Y., Spencer K., Jeyasekharan A.D., Sundar R., Goh B.C., Soo R. (2021). Low-dose pembrolizumab in the treatment of advanced non-small cell lung cancer. Int. J. Cancer.

[25] Renner A., Burotto M., Rojas C. (2019). Immune Checkpoint Inhibitor Dosing: Can We Go Lower Without Compromising Clinical Efficacy?. J. Glob. Oncol..

[26] Herbst R.S., Garon E.B., Kim D.-W., Cho B.C., Gervais R., Perez-Gracia J.L., Han J.-Y., Majem M., Forster M.D., Monnet I. (2021). Five year survival update from KEYNOTE-010: Pembrolizumab versus docetaxel for previously treated, programmed death-ligand 1–positive advanced NSCLC. J. Thorac. Oncol..

[27] Eisenhauer E.A., Therasse P., Bogaerts J., Schwartz L.H., Sargent D., Ford R., Dancey J., Arbuck S., Gwyther S., Mooney M. (2009). New response evaluation criteria in solid tumours: Revised RECIST guideline (version 1.1). Eur. J. Cancer.

[28] Schwartz L.H., Litière S., De Vries E., Ford R., Gwyther S., Mandrekar S., Shankar L., Bogaerts J., Chen A., Dancey J. (2016). RECIST 1.1—Update and clarification: From the RECIST committee. Eur. J. Cancer.

[29] Huang Y., Shen A. (2020). The prediction potential of neutrophil-to-lymphocyte ratio for the therapeutic outcomes of programmed death receptor-1/programmed death ligand 1 inhibitors in non-small cell lung cancer patients: A meta-analysis. Medicine.

[30] Jin J., Yang L., Liu D., Li W. (2020). Association of the neutrophil to lymphocyte ratio and clinical outcomes in patients with lung cancer receiving immunotherapy: A meta-analysis. BMJ Open.

[31] Faul F., Erdfelder E., Lang A.-G., Buchner A. (2007). G* Power 3: A flexible statistical power analysis program for the social, behavioral, and biomedical sciences. Behav. Res. Methods.

[32] Wu S., Wang L., Li W., Chen B., Liu Y., Wang H., Zhao S., Ye L., Sun H., He Y. (2020). Comparison between the first-line and second-line immunotherapy drugs in the progression-free survival and overall survival in advanced non-small cell lung cancer: A systematic review and meta-analysis of randomized controlled trials. Ann. Palliat. Med..

[33] Yoo S.H., Keam B., Kim M., Kim S.H., Kim Y.J., Kim T.M., Kim D.W., Lee J.S., Heo D.S. (2018). Low-dose nivolumab can be effective in non-small cell lung cancer: Alternative option for financial toxicity. ESMO Open.

[34] Wang Z., Zhan P., Lv Y., Shen K., Wei Y., Liu H., Song Y. (2019). Prognostic role of pretreatment neutrophil-to-lymphocyte ratio in non-small cell lung cancer patients treated with systemic therapy: A meta-analysis. Transl. Lung Cancer Res..

[35] Yang T., Hao L., Yang X., Luo C., Wang G., Lin Cai C., Qi S., Li Z. (2021). Prognostic value of derived neutrophil-to-lymphocyte ratio (dNLR) in patients with non-small cell lung cancer receiving immune checkpoint inhibitors: A meta-analysis. BMJ Open.

[36] Russo A., Russano M., Franchina T., Migliorino M.R., Aprile G., Mansueto G., Berruti A., Falcone A., Aieta M., Gelibter A. (2020). Neutrophil-to-lymphocyte ratio (NLR), platelet-to-lymphocyte ratio (PLR), and outcomes with nivolumab in pretreated non-small cell lung cancer (NSCLC): A large retrospective multicenter study. Adv. Ther..

[37] Bagley S.J., Kothari S., Aggarwal C., Bauml J.M., Alley E.W., Evans T.L., Kosteva J.A., Ciunci C.A., Gabriel P.E., Thompson J.C. (2017). Pretreatment neutrophil-to-lymphocyte ratio as a marker of outcomes in nivolumab-treated patients with advanced non-small-cell lung cancer. Lung Cancer.

[38] Mezquita L., Auclin E., Ferrara R., Charrier M., Remon J., Planchard D., Ponce S., Ares L.P., Leroy L., Audigier-Valette C. (2018). Association of the Lung Immune Prognostic Index With Immune Checkpoint Inhibitor Outcomes in Patients With Advanced Non-Small Cell Lung Cancer. JAMA Oncol..

[39] Gabrilovich D.I., Ostrand-Rosenberg S., Bronte V. (2012). Coordinated regulation of myeloid cells by tumours. Nat. Rev. Immunol..

[40] Diakos C.I., Charles K.A., McMillan D.C., Clarke S.J. (2014). Cancer-related inflammation and treatment effectiveness. Lancet Oncol..

[41] Mantovani A., Allavena P., Sica A., Balkwill F. (2008). Cancer-related inflammation. Nature.

